# Induced epileptic seizures in larval zebrafish reveal a synaptic modulation associated with the post-ictal state

**DOI:** 10.1093/braincomms/fcag323

**Published:** 2026-09-08

**Authors:** Dor Meron, Yarden Levinsky, Milagros Prendes, Inbar Brosh, Moti Freiman, Limor Freifeld

**Affiliations:** Biomedical Engineering Department, Technion—Israel Institute of Technology, Haifa 32000, Israel; Biomedical Engineering Department, Technion—Israel Institute of Technology, Haifa 32000, Israel; Biomedical Engineering Department, Technion—Israel Institute of Technology, Haifa 32000, Israel; Biomedical Engineering Department, Technion—Israel Institute of Technology, Haifa 32000, Israel; Biomedical Engineering Department, Technion—Israel Institute of Technology, Haifa 32000, Israel; Biomedical Engineering Department, Technion—Israel Institute of Technology, Haifa 32000, Israel

**Keywords:** epilepsy, larval zebrafish, neural activity imaging, expansion microscopy, glycinergic synapses

## Abstract

Epilepsy patients suffer from spontaneous and recurrent seizures. In some cases, after seizures, a transient post-ictal state associated with cognitive deficits develops. Moreover, seizure-induced alterations in brain structure and function may give rise to neurological comorbidities associated with epilepsy. Nevertheless, the mechanisms underlying neurological comorbidities in epilepsy broadly and the post-ictal state specifically are not well understood. Here we used a well-established model of acutely induced seizures in larval zebrafish to reveal how seizures modulate a neural circuit implementing a specific sensorimotor transformation, the escape response. Using *in vivo* calcium imaging, we found that the responsivity of Mauthner cells, hindbrain command-like neurons that normally evoke fast and strong escape responses, to startling stimuli was reduced following seizures. Moreover, we observed a global reduction in post-seizure responsivity of neurons to the stimuli and in spontaneous neural activity. To identify structural correlates of these changes, we used expansion microscopy to characterize synaptic inputs to the Mauthner cells. Using this approach, we discovered that seizures increased the density of receptors in glycinergic inhibitory synapses onto these cells, expected to increase synaptic strength. This enhancement may compensate for the lack of GABAergic inhibition during the seizure. In addition, inducing the disassembly of glycinergic synapses by post-seizure strychnine treatment, reversed the effect of reduced neural responsivity. Put together, these data show that, at both the synaptic and circuit levels, inhibitory drive is strengthened after seizure termination, giving rise to a post-ictal state similar to that defined in humans. Consequently, sensory sensitivity is reduced, and post-seizure behavioural deficits are expected. These results reveal a potential target for pharmacological interventions that may mitigate neurological deficits in epilepsy when the seizures themselves cannot be prevented.

## Teaser

Meron *et al*. report that the post-ictal state following pentylenetetrazole-induced seizures in larval zebrafish involves reduced neural responsivity to startling stimuli. Concurrently, receptor densities at inhibitory glycinergic synapses increase, and their disruption restores neural responsivity. This shows that glycinergic inhibition plays a critical role in mediating post-ictal sensory-motor deficits.

## Graphical Abstract

**Graphical Abstract:**
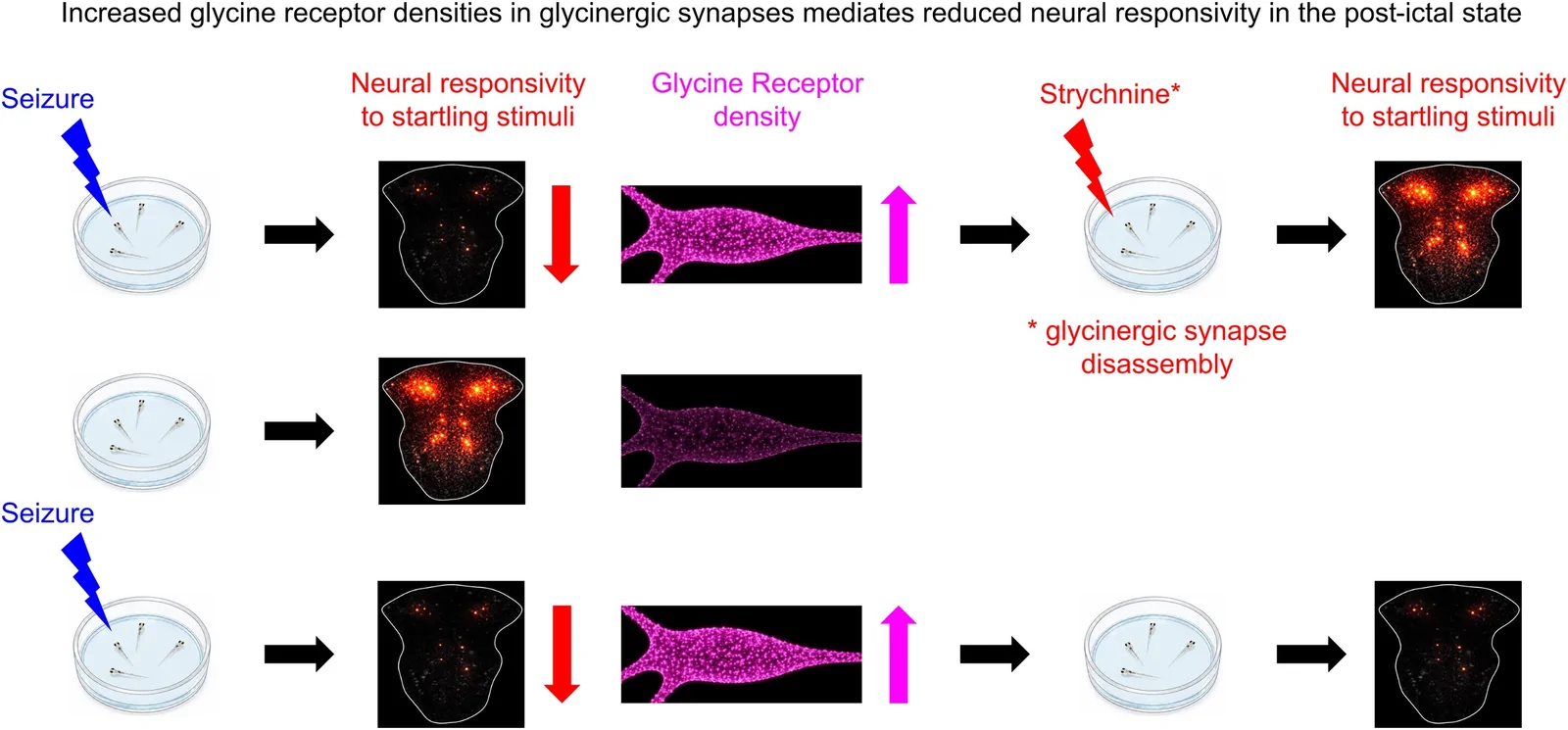

## Introduction

Epilepsy is a common neurological disorder affecting ∼1% of the global population. Epilepsy patients exhibit spontaneously recurring seizures—episodes of elevated and synchronous neural activity,^1,2^ which are drug-resistant in ∼30% of patients.^3,4^ Beyond seizures, epilepsy patients often present additional neurological comorbidities, including deficits in attention, learning and memory.^5-7^ In some cases, seizures lead to a post-ictal state lasting hours to days, associated with reduced activity measured via electroencephalography (EEG) as well as sensory, motor and cognitive deficits.^8,9^ Reduced perfusion or oxygenation were suggested as mechanisms underlying the post-ictal state^10^ while more broadly, neurological deficits in epilepsy are attributed to large-scale structural and network-level functional brain modulations.^11^ Epilepsy comorbidities are thought to arise from both the effects of epilepsy-causing mutations as well as short- and long-term effects of excessive neural activity during seizures, and seizure occurrence can also impact disease progression.^12-15^ Since all factors co-exist in humans, disentangling the specific mechanisms underpinning each of the various neurological deficits seen in epilepsy patients remains challenging.

Animal models of acutely induced seizures are advantageous for studying mechanisms underlying neurological deficits in epilepsies, as neurological deficits are absent prior to seizure occurrence and can thus be attributed to the impact of seizures on brain structure and function.^16-19^ However, past studies principally focused on models of temporal lobe epilepsies affecting the hippocampus. Thus, how neural structure and function are modulated in other brain areas and epilepsy models is less understood.

The larval zebrafish recently emerged as a powerful epilepsy model given its amenability to high-throughput drug screening and non-invasive *in vivo* imaging of neural activity.^20-25^ In particular, larval zebrafish exposed to pentylenetetrazole (PTZ), a γ-aminobutyric acid (GABA) A receptor antagonist,^26,27^ have been used as a generalized seizure model, and present seizure-like behaviours and epileptiform neural activity that are ameliorated by commonly used anti-seizure drugs.^28-37^ The acute effects of PTZ on larval zebrafish have been thoroughly characterized and are consistent with findings in both adult zebrafish and rodents.^38-43^

While the phenotypes of PTZ-induced seizures in larval zebrafish were extensively characterized, post-seizure effects on neural structure and function are poorly understood. Here, we reveal that after undergoing seizures induced by PTZ, larval zebrafish display reduced neural responsivity to startling stimuli.^44^ In particular, the rate at which Mauthner (M) cells, a pair of command-like hindbrain neurons mediating fast escape responses,^45^ responded to stimuli was dramatically reduced after seizures. This corresponds to a reduction in the rate at which fish decide to rapidly escape. Moreover, this reduction was accompanied by a broader decrease in neural responses to these stimuli as well as decreased spontaneous activity. Using Expansion Microscopy (ExM,^46,47^), we further identified a synaptic structural correlate of this functional modulation: An increased receptor density in glycinergic inhibitory synapses on the M cells within the same time window. Interestingly, inhibition of these receptors, known to cause their disaggregation from the synapse, reversed the post-seizure effect, in support of a causal role for glycinergic synapse enhancement in mediating reduced neural activity in response to stimuli after seizures. As the reduction in circuit activity that we observed is also consistent with symptoms of the post-ictal state in humans, these findings together suggest that enhancement of inhibition via synaptic plasticity may constitute one mechanism underlying this phenomenon.

## Materials and methods

### Zebrafish husbandry

Adult zebrafish were raised and maintained according to standard procedures. All experiments were performed on 6 days post-fertilization (dpf) larval zebrafish from the Tübingen long-fin strain, according to protocols approved by the Technion—Israel Institute of Technology animal care and use committee (IACUC, protocol number IL0820524). Larvae were raised in an incubator at 28.5°C with a 14-h light/10-h dark cycle.

### Calcium imaging of neuronal activity in response to startling stimuli

Calcium (Ca^2+^) imaging experiments were performed on individual pigment-free 6 dpf nacre^−/−^  *Tg(elavl3:H2B-GCaMP6s)jf5* larval zebrafish,^48^ in which the sensitive Ca^2+^ indicator GCaMP6s is expressed in the nuclei of all neurons. Experiments were conducted on larvae from seven distinct clutches. Experimental larvae and controls were assigned at random and both were included in each experimental day. First, larvae were immersed for 1 h in either 15 mM PTZ (experimental group; diluted from a 80 mM stock made from powder, Sigma-Aldrich, P6500) or embryo (E3) medium (controls) in the incubator. Then, the larvae were washed twice in E3 and incubated for 30 min. In experiments where PTZ treatment was followed by strychnine application, the incubation was replaced with immersion in 300 μM strychnine for 1 h, or E3 medium (in the corresponding group in which a seizure was induced but strychnine was not applied), then washed twice in E3 and transferred to imaging. Thus, in one experimental group larvae in which seizures were induced by PTZ were imaged 40 min post-seizure, and in two other experimental groups, they were imaged 75 min post-seizure (times take into account washout durations and handling required prior to imaging), either with or without strychnine treatment. To prevent movement during imaging, larvae were embedded within small petri dishes, dorsal side up, in 2% ultra-low melting point agarose (A2576-5G, Sigma-Aldrich) applied at 30°C. The agarose was allowed to solidify by slight cooling. Imaging was performed on an upright spinning-disk confocal microscope (″VIVO″, Intelligent Imaging Innovations, 3i), equipped with a scientific complementary metal-oxide-semiconductor (sCMOS) camera (Prime BSI, Photometrics) using a 20×, 1.0 numerical aperture (NA) objective, 488 nm laser excitation and a 24 msec exposure time. Using a piezo-objective scanner, five imaging planes, separated by 7 μm and surrounding the M cells, were sequentially scanned over time, achieving a per-plane sampling interval of 706 msec. For each larva, we conducted 10 experimental trials at 2-min inter-trial intervals to minimize habituation, capturing 250 frames per plane. Since the first 50 frames include the response to the visible-light laser used for imaging, analysis was conducted on the final 200 frames of each trial. Tap stimuli were triggered by the Slidebook software which was also used to control the time-series acquisition. The taps were applied to the microscope stage using an Arduino-controlled solenoid, similarly to^49^, at frame 100 of each trial.

### Analysis of neural activity in response to startling stimuli

Data from all trials were concatenated using an imageJ macro and analysed together. Motion artifacts were corrected using the ImageJ plugin ‘align slices in stack’.^50^ Subsequent analyses were performed in MATLAB. We manually applied a mask to restrict analysis to the hindbrain. Using CaImAn^51^ within the EZCalcium framework,^52^ we segmented individual cell nuclei and extracted their fractional fluorescence intensity changes (dF/F) over time, representing intracellular Ca^2+^ concentrations and thus neural activity. Because of its importance for data interpretation, we examined the raw movies and extracted fluorescence traces of M cells to determine in which trials stimulus responses occurred. To avoid double-counting the activity of neurons appearing in two neighbouring planes, we identified such neurons according to the proximity of their region of interest (ROI) centres and their highly correlated activity, and excluded the ROI associated with a lower signal-to-noise ratio. Finally, we defined a neuron as responsive to the stimulus in a trial when the ratio of the mean dF/F value in the 10 frames following the stimulus to the mean value in the 10 frames preceding the stimulus was larger than 1.5 times the standard deviation of the noise in the dF/F signal for that trial. With this definition, we computed the number of stimulus responsive neurons in each trial. Finally, to assess overall neural activity rates, we defined prominent peaks in the dF/F signals as activity transients and computed the number of transients per neuron, and the mean transient number across all neurons identified in each larva.

### Expansion microscopy of M cell glycinergic synapses

Experiments were conducted on 6 dpf wild-type larvae from five distinct clutches. As in activity imaging experiment, larvae were immersed in either fresh E3 (controls) or 15 mM PTZ (experimental group) for 1 h in the incubator, washed twice in E3 and incubated for 30 additional minutes. Experimental larvae and controls were assigned at random, and both were included on each experimental day. Subsequently, the larvae were euthanized, and fixed overnight at 4°C in 4% Paraformaldehyde (PFA; Rhenium, TS-28906), diluted from a 16% stock, in phosphate-buffered saline (PBS; Fisher Scientific,70011036). Then, the fixative was washed out in 1× PBS with 0.25% Triton-X five times for 5 min, and the brains were dissected out.

We followed the protocol we previously developed for staining glycinergic synapses and applying ExM to larval zebrafish brains.^46,47^

To stain glycine receptors (GlyRs), we used standard immunohistochemistry procedures, which we have previously applied for this purpose.^46,53^ Accordingly, larval brains were incubated in antigen retrieval medium (150 mM Tris-HCl pH 9.0; Sigma-Aldrich, T2819) for 5 min at room temperature and 15 min at 70°C, and then permeabilized in 0.05% Trypsin-Ethylenediaminetetraacetic acid (Trypsin-EDTA; Biological Industries, Israel Beit-Haemek Ltd., 03-052-1B) for 40 min on ice. Then, the brains were placed in a block solution containing 1× PBS, 0.25% Triton X (Sigma-Aldrich, 93443), 1% bovine serum albumin (Sigma-Aldrich, A3059), 5% normal goat serum (Sigma-Aldrich, G9023) and 1% dimethyl sulfoxide (DMSO; Sigma-Aldrich, D8418) for 2 h at 4°C. Primary and secondary antibodies were each diluted 1:200 in block solution and applied for 72 h at 4°C. Between steps, brains were washed three times in 1× PBS with 0.25% Triton-X. We used the primary antibodies anti-GlyR (146008, Synaptic Systems) and anti- Synaptic Vesicle Protein 2 (SV2, Developmental Studies Hybridoma Bank), and the secondary antibodies Alexa 546 goat anti-rabbit (Invitrogen, A1107) and Atto647N goat anti-mouse (Sigma-Aldrich, 40839).

To apply ExM to larval zebrafish brains, we used a protocol we previously adapted for this purpose.^46,47^ Thus, brains were anchored with 0.1 mg/ml 6-((acryloyl)amino)hexanoic acid, succinimidyl ester (Acryloyl-X, SE; Thermo Scientific, A20770) diluted in 1× PBS with 0.5% Triton-X. The gel composition was as described in.^46,47^ A silicon isolator (Sigma-Aldrich, GBL664501) served as a gelling chamber, with 40 μl of gelling solution applied per sample. Gel monomers were allowed to diffuse into the tissue for 45–60 min at 4°C, then polymerized in a humid environment for 2–2.5 h at 37°C. Following gelation, samples were digested in 16 units/ml Proteinase K (New England Biolabs, P8107S), diluted 1:50 from a stock of 800 units/ml in 50 mM Tris, 25 mM EDTA and 0.5% Triton-X at 50°C, overnight in a humid environment. Then, the digestion buffer was replaced with 1 × PBS, and the gels were kept at 4°C until expansion in a large volume of doubly deionized water. The water was replaced every 20 min, 3–5 times, until full expansion was reached. Gels were placed on a petri dish pretreated with 0.1% (w/v) Poly-L-Lysine solution (Sigma-Aldrich, p8920) for 20 min to ensure attachment of the gels to the plate, minimizing motion artefacts during imaging. To compute sample expansion factors, brains were imaged on an Olympus MVX10 wide-field macro-zoom fluorescent microscope at a 10× magnification prior to gelation and after expansion. The expansion factor for each brain was estimated as the mean of the ratios of five measurements of distances between identifiable brain features in pre- and post- expansion images.

Expanded gels were imaged on an upright spinning-disk confocal microscope (″VIVO″, Intelligent Imaging Innovations, 3i), using a 40×, 1.0 NA objective, with 560 nm laser excitation and a mean exposure time of 464 msec (range 400–700) for GlyR imaging and with 640 nm laser excitation and a mean exposure time of 423 msec (range 250–700) for SV2. Expanded M cells typically spanned two imaging tiles and ∼200 frames at a z-step of 0.59 μm—set according to the microscope’s resolution limit.

### Analysis of ExM images of glycinergic synapses on the M cells

Acquired images underwent deconvolution in Slidebook software, followed by pairwise stitching^54^ and background and shading correction (when required) using BaSiC.^55^ To generate a segmentation mask covering the M cell cytoplasm, we applied the ‘morphological geodesic active contour’ algorithm^56^ to the SV2 image stack down-sampled to an equal resolution in all axes (i.e. increasing the x, y pixel dimensions from 0.1625 to 0.59 μm). Bilateral and inverse Gaussian filtering were applied (in Python) to smooth the image and enhance the edges prior to generating the mask. Typically, multiple masks were produced and stitched together to cover the entire M cell. Finally, the mask was up-sampled to the original image resolution, and dilated (in MATLAB) so as to also cover the M cell membrane. M cells for which a mask could not be fitted, or that were accidentally not imaged in full, were excluded from subsequent analysis (2 M cells in experimental larvae, 4 M cells in controls).

Subsequent analyses were performed in MATLAB, on the GlyR image stack up-sampled to an equal resolution in all axes (i.e. reducing the z pixel dimensions from 0.59 to 0.1625 μm). Within the M cell mask, a binary image of pixels containing GlyRs was generated by applying a bilateral filter for smoothing and hysteresis-based segmentation. The two intensity thresholds for hysteresis were set to the mean signal within the M cell membrane mask (generated by subtracting the original M cell cytoplasm mask from the dilated mask) plus 1 and 2 standard deviations, respectively. Then, we quantified the extent to which the membrane is covered by glycinergic synapses as the fraction of segmented receptor pixels out of all M cell membrane pixels. We also quantified the intensity in GlyR pixels, the intensity in other membrane pixels (potentially containing extra-synaptic receptors) and the 95th percentile of GlyR signal intensity within the M cell cytoplasm (representing the staining of intracellular receptors not yet transported to the membrane). With these metrics, we assessed the receptor density in synapses and corrected for any potential effect of staining quality or imaging conditions that can vary between samples. Finally, to compare signal intensities in the soma and the lateral dendrite, we visually identified the change in curvature signifying the soma-to-dendrite transition in each image dataset and divided the data accordingly.

To quantify the levels of the presynaptic Synaptic-Vesicle protein 2 (SV2) in glycinergic synapses, we applied the GlyR pixels mask, the M cell mask, and the M cell mask excluding GlyR pixels, and computed average SV2 signal intensities in these masks. In addition, using dilation and elimination of the original GlyR pixels, we generated a mask of the GlyR surroundings, and quantified the SV2 signal in this mask as well as in a mask of the rest of the membrane, which excluded both the GlyR pixels and the pixels surrounding them.

### Statistical analysis

Prior to performing statistical hypothesis tests, the normality of the distribution of values in each experimental or control group was tested via an Anderson-Darling goodness-of-fit hypothesis test. If normality was not excluded for most groups, a parametric test was used, and otherwise, a non-parametric test was used. Thus, comparisons of one experimental group to one control group were conducted using unpaired two-tailed t-tests without assuming equal variances when normality was not excluded, and with a Wilcoxon rank-sum test when it was excluded. For the comparison of dendritic and somatic GlyR intensity in the same cells, a paired *t*-test was used. Similarly, when normality was not excluded, multiple groups were compared with an ANOVA analysis by post-hoc tests using Tukey’s honestly significant difference procedure to identify which specific group means significantly differed. Otherwise, we used a Kruskal-Wallis test, followed by multiple comparisons via the Dunn-Šidák method. When comparing mean intensities in the somas and dendrites of M cells within the control and experimental groups, we used the non-parametric Wilcoxon signed-rank test for paired samples in both groups, since the distribution of values in the experimental group significantly deviated from normality.

## Results

### M cell responses—initiating a fast escape behaviour—are reduced after acutely induced seizures

**Figure 1:**
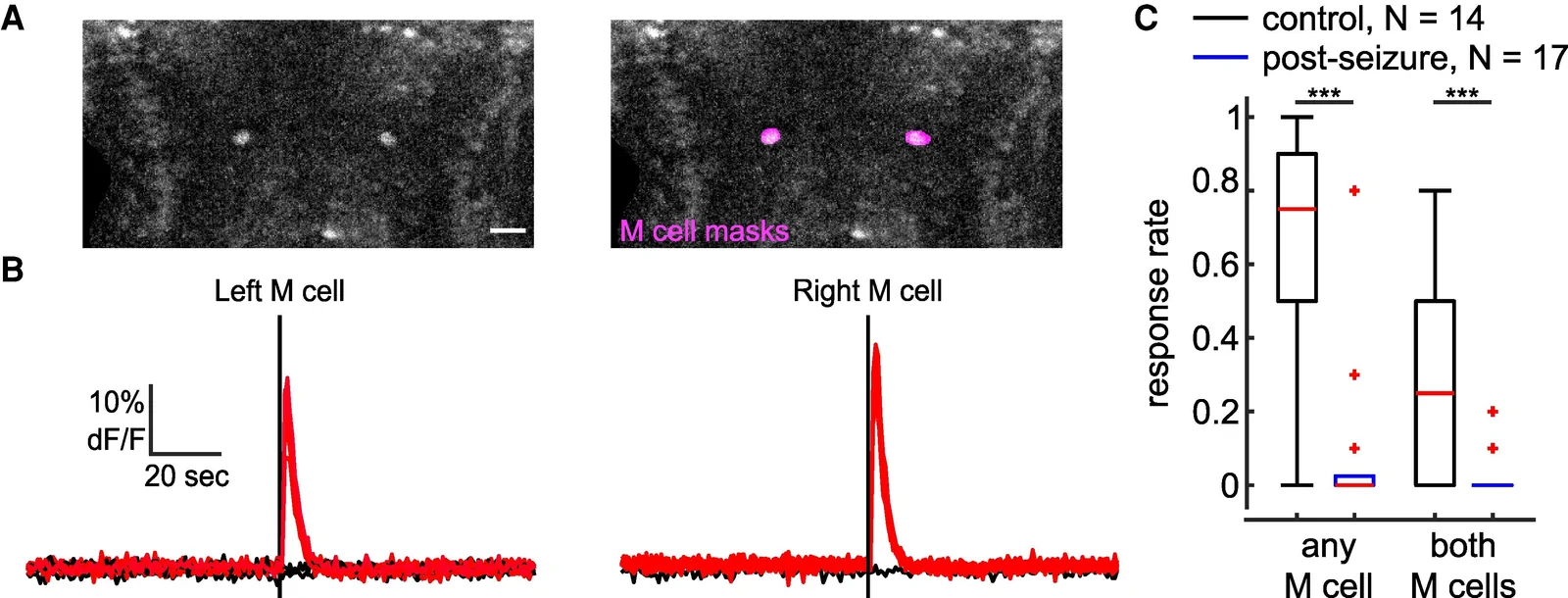
**M cell responses to startling stimuli are reduced after acutely induced seizures.** (**A**) Left—Maximal intensity projection of a time series from one trial in a control *Tg(elavl3:H2B-GCaMP6s)jf5* 6 dpf larval zebrafish, showing the hindbrain area surrounding the two M cell nuclei, easily identifiable according to their location and size. Scale bar—10 μm. Right—same image as on the left, overlaid with the masks of the M cells, hindbrain neurons mediating fast escape responses, identified via analysis with CaImAn. (**B**) Activity (measured as fractional fluorescence intensity over time, dF/F) of the identified M cells during 10 distinct trials. Red traces—trials in which the neuron responded to the stimulus; Black traces—no-response trials. The Left M cell responded in 8 of the 10 trials and the right M cell in 9 of the 10 trials, such that neither cell responded in the first trial. As expected, the M cells are not active except in response to the stimulus. (**C**) Box plots of the fraction of trials in which a response in any of the M cells (left two columns), or both of them (right two columns) was observed, in controls (black boxes, *N* = 14 fish) or in experimental larvae that previously underwent a seizure induced by 15 mM PTZ (blue boxes, *N* = 17 fish). Here and in all box plot figures: Red line—median; bottom and top edges—25th and 75th percentiles, respectively; whiskers extend to the most extreme data points not considered as outliers. Red ‘+’ marks indicate outliers. *** *P* < 0.001 according to two-tailed Wilcoxon rank-sum tests, adjusted with a Holm-Bonferroni correction to control the family-wise error rate.

To determine whether and how sensory processing is altered after acutely induced seizures, we exposed 6 dpf *Tg(elavl3:H2B-GCaMP6s)jf5* larval zebrafish, expressing the Ca^2+^ indicator GCaMP6s in the nuclei of all neurons, to 15 mM PTZ for 1 h. Thirty minutes after washout, we applied 10 strong acoustic startling stimuli (taps applied to the stage^49^) at ∼2 min intervals, preventing habituation,^57,58^ and captured neural activity in the hindbrain region containing the escape circuit motifs.^59^ First, we examined M cell responses (Fig. 1A, B), as their activation reflects the initiation of a fast and strong escape response (a short-latency C-start, SLC^60^). As expected, control larvae (*N* = 14) showed a high M cell response rate (Fig. 1C): At least one of the two M cells was active in 65 ± 8.6% of trials (mean ± standard error of the mean across fish), and both responded in 29.3 ± 7.45% of trials—consistent with the lack of directionality of the tap stimulus and past reports.^61^ In contrast, larvae previously treated with PTZ (*N* = 17) exhibited dramatically reduced response rates: The mean fraction of trials in which any of the M cells responded was only 7.7 ± 4.9%, with both M cells responding in only 1.76 ± 1.3% of trials (z = 4.11, *P* = 7.9015 × 10^−5^ and z = 3.49, *P* = 4.9117 × 10^−4^, respectively; two-tailed Wilcoxon rank-sum test and a Holm-Bonferroni correction applied to control the family-wise error rate). In addition, while M cell responses occurred in 13 of 14 control larvae, only 4 of 17 PTZ-treated larvae showed any M cell responses. Together, these results demonstrate that seizures strongly reduce the responsiveness of larval zebrafish to startling sensory inputs.

### Neural activity is reduced after acutely induced seizures

**Figure 2:**
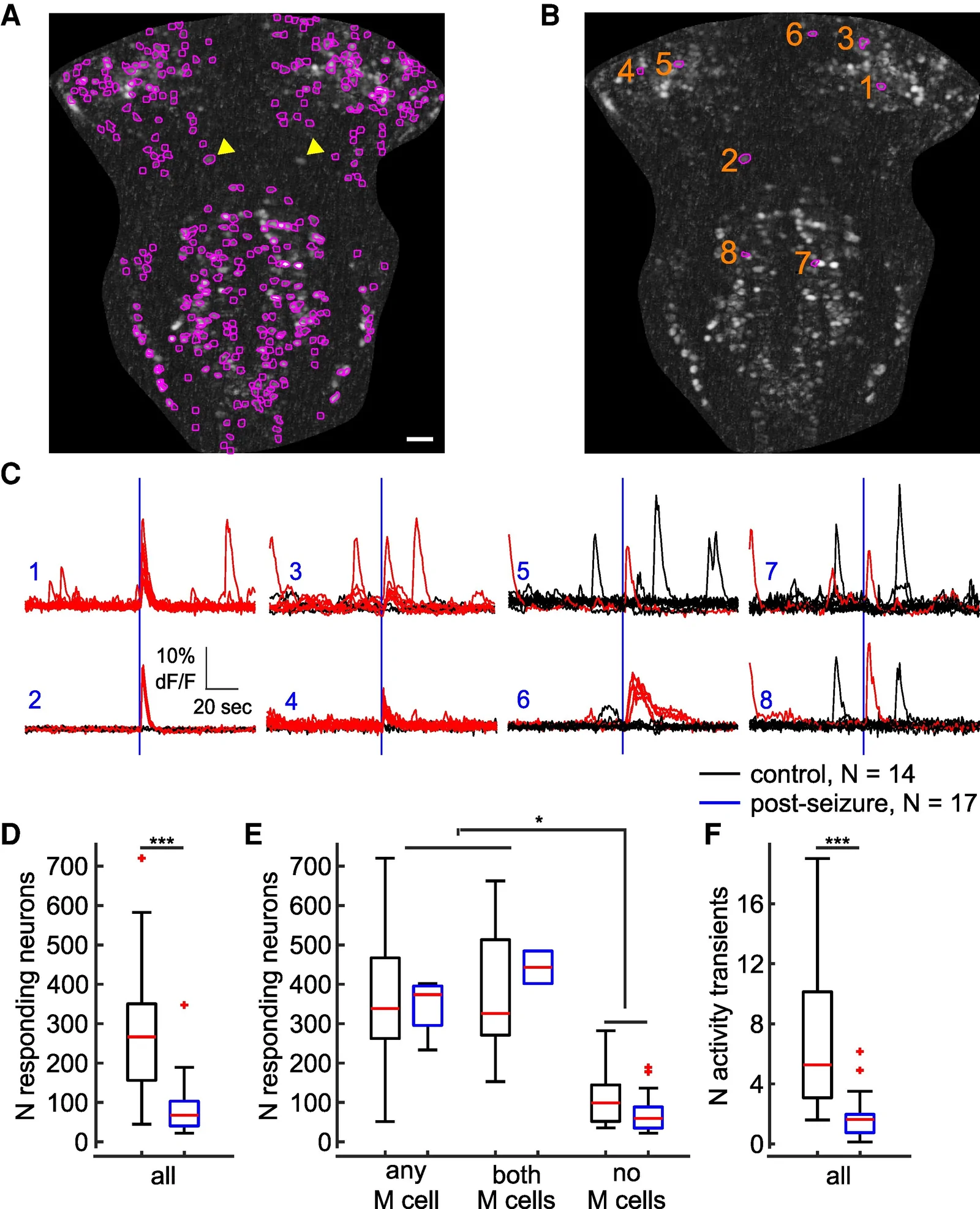
**Neural responses to startling stimuli are low after acutely induced seizures.** (**A**) Identified regions of interest (contours) overlaid on a map of activity in the hindbrain of a 6 dpf *Tg(elavl3:H2B-GCaMP6s)jf5* larval zebrafish, generated by CaImAn. Higher intensities indicate high activity. Arrowheads point to the identified nuclei of the M cells. Only one was assigned a ROI in the presented plane; the other M cell was assigned a ROI in an adjacent plane where its signal was stronger (see methods). Scale bar—10 μm. (**B**) Contours of a subset of neurons, whose activity in response to startling stimuli is presented in C. (**C**) Neural activity (normalized fluorescence intensity changes over time—dF/F) in 10 overlaid trials. Red traces—trials in which the neuron responded to the stimulus; Black traces—no-response trials. Stimulus application times are indicated by vertical blue lines. (**D**) Box plots of the number of hindbrain neurons that responded to the stimulus in control (black boxes) and experimental larvae, in which a seizure was previously acutely induced (blue boxes). *** *P* < 0.001 according to a two-tailed Wilcoxon rank-sum test. (**E**) Box plots of the number of hindbrain neurons that responded to the stimulus, in control and experimental larvae, divided by whether or not any (left), both (middle) or no (right) M cells were active in the corresponding trials. Colours as in (**D**). A two-way ANOVA analysis showed a significant effect of M cell responsivity on the number of responding neurons (F = 28.61; degrees of freedom—df = 2,51; *P* = 4.6619*10^−9^) and not on the post-seizure versus control condition (F = 0.01; df = 1,51; *P* = 0.9358). * *P* < 0.05 in all pairwise comparisons, using Tukey’s honestly significant difference procedure, between the two groups where no M cell responses were observed and the other four groups. (**F**) Box plots of the mean number of activity transients across all neurons in control and experimental larvae *** *P* < 0.001 according to a two-tailed Wilcoxon rank-sum test colours as in (D, E).

Having observed reduced M cell responsivity to startling stimuli after seizures, we next asked whether this reduction reflects lower stimulus responsivity throughout the startle circuit. Using CaImAn,^51^ we assigned regions of interest (ROIs) to all imaged hindbrain neurons and extracted their activity traces (Fig. 2A–C). We defined stimulus-responsive neurons as those showing a significant fluorescence increase following the stimulus application (see Methods). The total number of responsive neurons in control larvae, summed across all imaged hindbrain planes, 302 ± 51, was significantly higher than in experimental larvae, 89 ± 19 (z = 3.67, *P* = 2.4093 × 10^−4^, two-tailed Wilcoxon rank-sum test; Fig. 2D). Together with the decrease in M cell stimulus responses, this suggests reduced sensory sensitivity. However, since M cells are active when larvae decide to escape, and hindbrain activity shapes this behavioural response,^59,60,62^ the reduction in overall neural responsivity could be driven, at least in part, by the reduced rate at which larvae decided to rapidly escape.

To examine whether the number of stimulus-responsive neurons depends on M cell activity, we analysed neural responsivity separately for trials with and without M cell responses (Fig. 2E). A 2-way ANOVA analysis of the responsivity data with this division showed a significant effect only to the M cell responsivity (F = 28.61; degrees of freedom—df = 2,51; *P* = 4.6619 × 10^−9^), and not to the post-seizure versus control condition (F = 0.01; df = 1,51; *P* = 0.9358). In particular, using post-hoc multiple comparisons using Tukey’s honestly significant difference procedure, we found that, in control larvae, the mean number of responsive neurons was similar when both M cells were active (371 ± 50) and when at least one M cell was active (366 ± 53; q = 0.15, *P* = 1) and higher in both cases than the number of responsive neurons when no M cells were active (114 ± 21; q = 6.71, *P* = 2.4263 × 10^−4^ and q = 7, *P* = 1.1934 × 10^−4^, respectively). Interestingly, with this division of trials, the numbers of responsive neurons in post-seizure experimental larvae were comparable to the numbers in control larvae (443 ± 41 when both M cells were active, q = 1.06, *P* = 0.9749; 346 ± 39, when at least one was active, q = 0.4, *P* = 0.9997; and 73 ± 12 when no M cells were active, q = 1.21, *P* = 0.9547). Moreover, the number of responding neurons when no M cells were active was significantly different from the number of responding neurons when any or both M cells were active, also in post-seizure larvae, as in controls (q = 5.59, *P* = 0.0031 and q = 5.64, *P* = 0.0028, respectively). This suggests that the reduced responsivity of hindbrain neurons post-seizure is due to reduced sensory sensitivity leading to a low rate of decisions to rapidly escape, and that there’s no additional decrease in the recruitment of neurons mediating the motor response once a rapid escape is initiated.

If seizures have a long-lasting impact on neural activity, this effect is unlikely to be specific to startle responses. To test whether reduced neural responsivity to startling stimuli reflects a broader post-seizure effect on neural activity, we also analysed the rate of spontaneous neural activity transients throughout the recording, including before and after the stimuli. For this purpose, we quantified the number of prominent changes in the fractional GCaMP fluorescence (dF/F) for each neuron, and computed the mean rate of activity per larva. Interestingly, this analysis revealed a general post-seizure reduction in neural activity (Fig. 2F): The number of activity transients in control larvae (6.89 ± 1.32) was significantly higher than in experimental larvae (1.85 ± 0.39; z = 3.6361, *P* = 2.7685 × 10^−4^, Wilcoxon rank-sum test). Taken together, in this system, acutely induced seizures drive a global reduction in neural activity alongside reduced stimulus responsivity.

### Acutely induced seizures give rise to an increase in receptor densities at glycinergic synapses

We next sought to identify a synaptic correlate of these observed changes in neural activity. Inhibitory glycinergic synapses on the M cells play a key role in establishing the M cells sensitivity threshold to excitatory inputs, and thus their tendency to fire an action potential and initiate a short latency C-start escape (SLC).^63^ Accordingly, enhancement of these synapses could explain the reduced M cell response rate to startling stimuli, akin to the role such potentiation plays in lowering the tendency to escape during non-associative learning.^64,65^ Moreover, since PTZ blocks inhibitory GABAergic synapses, we hypothesized that glycinergic inhibitory synapses, unaffected by PTZ, would be strengthened to compensate for the excessive excitation caused by reduced GABAergic inhibition during the seizures.

**Figure 3:**
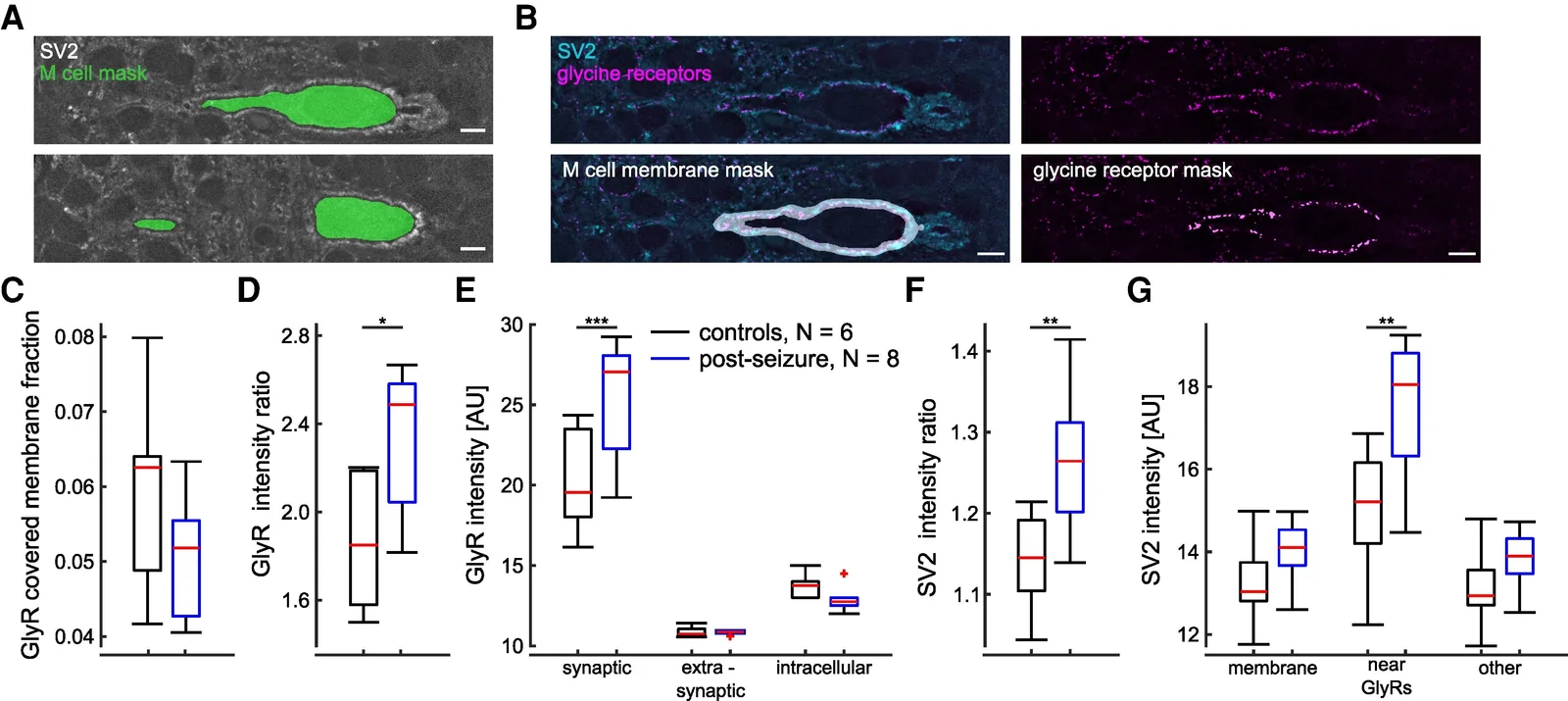
**Glycine receptors (GlyRs) in glycinergic synapses are more densely packed after seizures.** (**A**) Two planes from an M cell in an experimental fish, identifiable via surrounding Synaptic Vesicle protein 2 (SV2) staining (grey) and the characteristic shape of the axon cap covering the M cell axon initial segment. The image is down-sampled to have equal resolution in x-y and z, as this was used for M cell segmentation. Overlaid is a segmentation mask (green) of the M cell cytoplasm. Scale bars in all figure panels—5 μm (corrected for an estimated expansion factor of 3.77). (**B**) Top—Another plane from the same M cell as in A, showing both SV2 (cyan) and GlyR (magenta) staining (left), or GlyR staining only (right). Bottom—Same images as above, overlaid with a segmentation mask of the M cell membrane (left) and of the GlyRs within the membrane (right). (**C**) Box plots describing the fraction of the M cell membrane covered by GlyRs in control fish (black box) and experimental fish that have previously undergone a PTZ-induced seizure (blue box). Group means were compared with a two-tailed *t*-test with unequal variances and did not significantly differ (t(degrees of freedom—df = 7.73) = 1.54, standard error of the difference—SE = 0.0061, *P* = 0.1625). (**D**) Box plots describing the ratio of GlyR intensity in synapses (segmented as in the bottom right panel in B) and in the rest of the M cell membrane (segmented as in the bottom left panel in B), in controls (black box) and experimental fish (blue box). * *P* < 0.05 according to a two-tailed *t*-test with unequal variances (t(df = 11.2) = 2.82, SE = 0.1694). (**E**) Box plots describing the GlyR intensity in segmented synapses (left), the rest of the M cell membrane (middle) and intracellularly—within the M cell (right), for controls (black boxes) and experimental (blue boxes) fish. A two-way ANOVA analysis showed that both the receptor location (F = 126.16; df = 2,36) and the experimental condition (F = 4.99; df = 1,36) affect GlyR signal intensities. *** *P* < 0.001 in post-hoc multiple comparisons using Tukey-Kramer’s honestly significant difference procedure comparing GlyR signal intensities within synapses between control and experimental larvae (differences in other locations were not significant). (**F**) Box plots describing the ratio of SV2 intensity in the GlyR mask and in the rest of the M cell membrane, in controls (black box) and experimental fish (blue box). ** *P* < 0.01 according to a two-tailed *t*-test with unequal variances (t(df = 11.96) = 3.06, SE = 0.04). (**G**) Box plots describing the SV2 intensity in the entire M cell membrane (left), and divided into ‘near GlyRs’—in the GlyR mask and ‘other’—in the rest of the M cell membrane (middle and right, respectively), for controls (black boxes) and experimental (blue boxes) fish. As in E, a two-way ANOVA analysis showed a significant effect on SV2 intensity for both the location (F = 12.39; df = 1,36) as well as the experimental condition (F = 22.07; df = 2,36). ** *P* < 0.01 according to Tukey-Kramer’s multiple comparisons procedure following the two-way ANOVA analysis.

To test this, we induced seizures using 15 mM PTZ for 1 h and captured the spatial distribution of GlyRs on the M cells 30 min after washout, using the protein-retention ExM protocol adapted for zebrafish.^46,47,66^ Since antibody staining labels all GlyRs and not just ones on the M cell, we first segmented the M cell using Synaptic Vesicle protein 2 (SV2) staining, which highlights the M cell boundaries as it receives synaptic input throughout its membrane (^45^ Fig. 3A). Then, we segmented the GlyRs based on their staining along the M cell membrane, to quantify the extent to which GlyRs covered the membrane as well as the synaptic signal intensity, reflecting the density of receptors within the synapses (Fig. 3B). This analysis showed that acutely induced seizures modulate glycinergic inhibitory synaptic densities (Fig. 3C–E): While the fraction of the M cell membrane covered by receptors did not significantly differ between controls (0.06 ± 0.005, *N* = 6 fish; Fig. 3C) and post-seizure larvae (0.051 ± 0.003, *N* = 8; t = 1.54, df = 7.73, *P* = 0.1625), the ratio of the GlyR signal intensity in segmented glycinergic synapses to the baseline M cell membrane signal (which may include extra-synaptic GlyRs) was higher in experimental larvae (2.34 ± 0.114) compared to controls (1.86 ± 0.126; t = 2.82; df = 11.2; *P* = 0.0164; Fig. 3D). Since this ratio reflects the GlyR density in glycinergic synapses, this implies that PTZ-induced seizures give rise to an increase in receptor densities in inhibitory glycinergic synapses.

We note that since the GlyR mask was generated using intensity-based thresholds, the slightly higher M cell membrane fraction containing GlyRs that was identified in control fish (and was not statistically significant) likely also reflects a higher GlyR density in experimental fish, rather than a difference in synapse number or size. Additionally, since imaging exposure times were typically higher in controls (537.5 ± 53.2 msec) than in experimental larvae (421.4 ± 28.1 msec), to compensate for a signal intensity difference, imaging condition differences cannot explain the higher GlyR signal intensity observed post-seizure. Nevertheless, to further confirm that this difference was not due to variability in staining quality, we compared the synaptic GlyR signal intensity to both the extra-synaptic intensity in the rest of the M cell membrane and to the intensity of intracellular receptors stained by the antibody (Fig. 3E, the latter measured as the 95th percentile intensity of the cytoplasmic GlyR signal). A two-way ANOVA analysis of the results revealed a significant effect on the GlyR signal intensity to both the experimental condition (F = 4.99; df = 1,36; *P* = 0.0318) and, as expected, to the receptor location (F = 126.16; df = 2,36; *P* = 5.444 × 10^−17^). In particular, we found that the higher GlyR intensity ratio in experimental larvae compared to controls was due to a difference in the GlyR signal intensity within the segmented synapses (controls: 20.18 ± 1.34 [Arbitrary Units—AU], experiment: 25.4 ± 1.3; q = 6.5754, *P* = 5.8224 × 10^−4^ according to post-hoc multiple comparisons using Tukey-Kramer’s honestly significant difference procedure). In contrast, extra-synaptic and intracellular intensities were similar in experimental and control larvae (extra-synaptic—controls: 10.85 ± 0.135 [AU], experiment: 10.85 ± 0.042 [AU]; q = 0.001, *P* = 1; intracellular—controls: 13.75 ± 0.31 [AU], experiment: 12.88 ± 0.26 [AU]; q = 1.1, *P* = 0.9693). Thus, the higher GlyR signal in glycinergic synapses in post-seizure larvae cannot be attributed to inter-sample differences in staining quality or imaging conditions. Together, these data indicate that seizures induce a GlyR signal intensity increase that represents elevated GlyR density in glycinergic synapses. Figure panels showing all data points associated with these results are included as Supplementary Fig. 1A–C.

### The post-synaptic increase in glycine receptor densities is accompanied by a presynaptic increase in the signal of synaptic vesicle protein 2

Having observed the above described increase in GlyR signal intensities after PTZ induced seizures, representing elevated GlyR densities, we next asked whether the enhancement of glycinergic synapses is also associated with pre-synaptic changes, and in particular with an increase in the levels of Synaptic Vesicle protein 2 (SV2). For this purpose, we also analysed the intensity of the pre-synaptic signal arising from the SV2 staining within the mask covering GlyRs (Fig. 3F). This revealed that the ratio of the SV2 signal within glycinergic synapses and outside these synapses, on the M cell membrane, similarly to the GlyR signal, was higher in the post-seizure group (1.26 ± 0.03) compared to the control group (1.14 ± 0.025, t = 3.06, df = 11.96, *P* = 0.0099 according to a two-tailed *t*-test with unequal variances). Further examining the SV2 signal separately, in the entire membrane, at the glycinergic synapses, and at the rest of the membrane (Fig. 3G), a 2-way ANOVA analysis revealed a significant effect to the region (all membrane versus intra- or extra-synaptic: F = 22.07; df = 2,36; *P* = 5.5541 × 10^−7^) as well as to the condition (controls versus post-seizure: F = 12.38; df = 1,36; *P* = 0.0012). Moreover, post-hoc multiple comparisons showed a significant difference in the synaptic SV2 signal (post-seizure fish: 17.51 ± 0.63; controls: 14.98 ± 0.68; q = 5.39, *P* = 0.0064) and no difference between the membranal SV2 signal in post-seizure fish (14.02 ± 0.26) and controls (13.23 ± 0.44; q = 1.7, *P* = 0.8342) as well as between the extra-synaptic SV2 signal in these populations (post-seizure fish: 13.83 ± 0.24; controls: 13.11 ± 0.42; q = 1.53, *P* = 0.8847). Figure panels showing all data points associated with these results are included as Supplementary Fig. 1D, E.

Since the resolution obtained via ExM generates a separation between the post-synaptic GlyR signal and the SV2 pre-synaptic signal, such that the latter is further away from the M cell soma centre than the former (Supplementary Fig. 2A), and since we wanted to make sure the SV2 signal increase was not generated by cross-talk between the imaging channels and a strengthened GlyR signal, we repeated the above analysis while using a mask that covers the surroundings of GlyRs within the M cell membrane and excludes the synaptic GlyR clusters. Indeed, the ratio of the SV2 signal near GlyRs, and in the rest of the membrane (excluding the GlyRs and their surroundings) was higher in the post-seizure group (1.14 ± 0.02) compared to controls (1.08 ± 0.01; t = 3.25, df = 11.77, *P* = 0.0072 according to a two-tailed *t*-test with unequal variances; Supplementary Fig. 2B). Moreover, a 2-way ANOVA analysis of intensities around GlyRs and in the rest of the membrane in controls and experimental fish, showed a significant effect to both the condition (F = 7.1; df = 1,24; *P* = 0.0136) as well as to the part of the membrane in which the SV2 signal was analysed (F = 16.62; df = 1,24; *P* = 4.3379 × 10^−4^). Post-hoc multiple comparisons following this analysis showed that the higher ratio in the post-seizure group was generated by a significantly higher signal intensity around GlyRs in the post-seizure group (15.2 ± 0.35) compared to controls (13.78 ± 0.48; q = 4.01, *P* = 0.0425), while the signal in the rest of the membrane was no different between the post-seizure group (13.28 ± 0.2) and controls (12.81 ± 0.39; q = 1.32, *P* = 0.7869; Supplementary Fig. 2C). Figure panels showing all data points associated with these results are included as Supplementary Fig. 2D, E.

Put together, this demonstrates that the enhancement of glycinergic synapses following PTZ-induced seizures involve both an increase in receptor densities as well as presynaptic strengthening.

### GlyRs are more dense in the M cell lateral dendrite than in the soma, and this difference is preserved following acutely induced seizures

**Figure 4:**
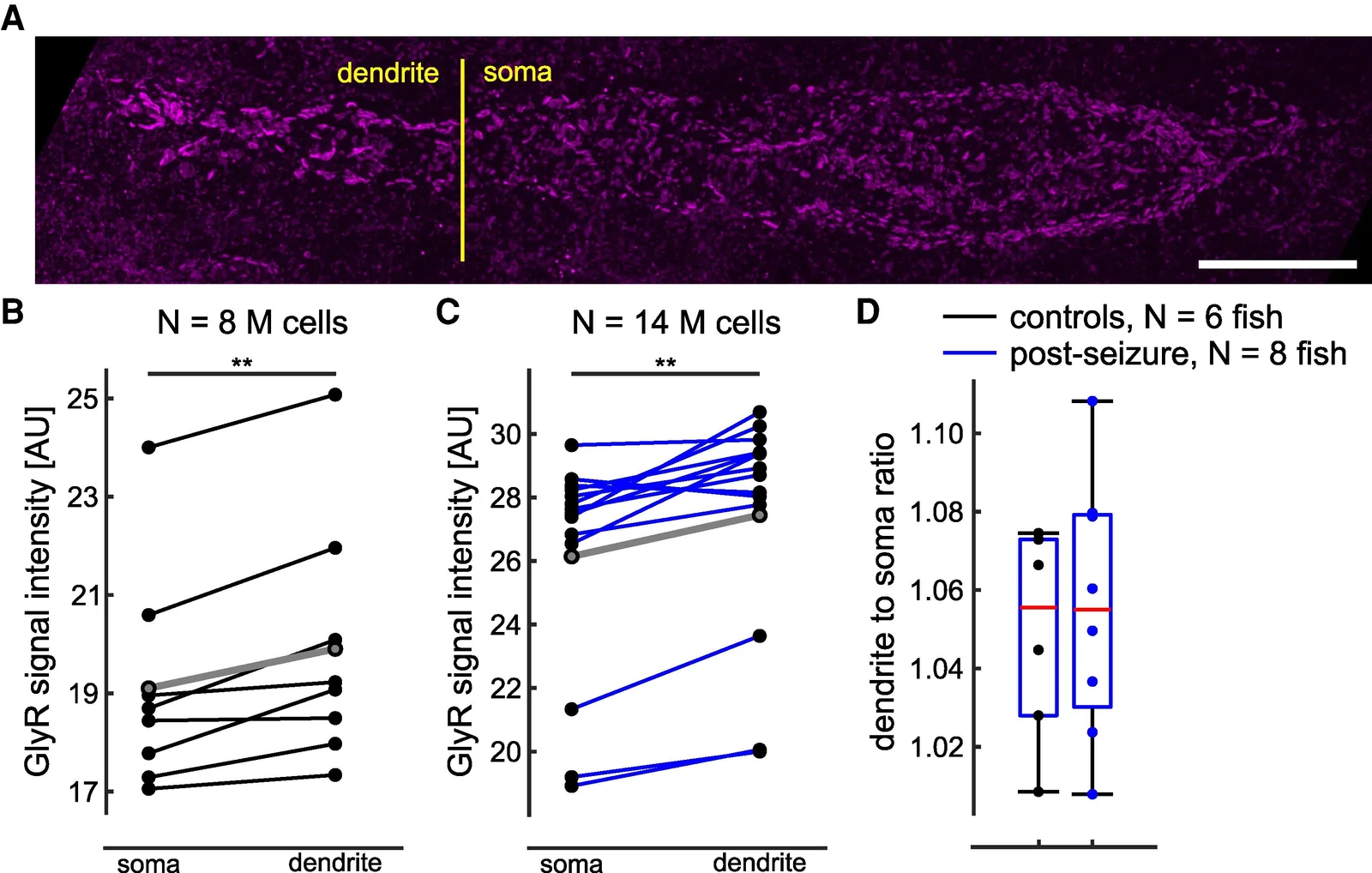
**Glycine receptor density is higher in M cell dendrites compared to the somas.** (**A**) Maximum intensity projection of an M cell from one example fish showing glycine receptors on the soma and dendrite. Yellow line—the boundary defined between the two cellular compartments and subsequently used for analysis. Scale bar—10 μm (corrected for an estimated expansion factor of 3.77). (**B**) A comparison of GlyR signal intensities in the soma (left) and dendrites (right) of individual M cells (*N* = 8) in control fish (*N* = 6). Black dots: soma/dendrite GlyR intensities. Black lines: the change in intensities between the soma and the dendrite of an M cell in a control fish. Grey dots and line: mean GlyR intensities in the soma and dendrite and the change between them. ** *P* < 0.01 according to a paired Wilcoxon signed-rank test (signed rank = 0). (**C**) A comparison of GlyR signal intensities in the soma and dendrites as in B, but for experimental fish (blue lines replace black lines; *N* M cells = 14; *N* fish = 8). ** *P* < 0.01 according to a paired Wilcoxon signed-rank test (signed rank = 5). (**D**) Box plots describing the ratio of dendrite to soma GlyR intensity in control (black box) and experimental (blue box) fish; same fish as in B, C. Dots—ratios of individual fish, overlaid on box plots. Group means were compared with two-tailed t-tests with unequal variances (t(df = 11.86) = 0.4, SE = 0.016, *P* = 0.6947).

While visually inspecting the data, we noticed that the GlyR signal intensity was not uniform throughout the M cell, but rather higher in the lateral dendrite than in the soma. To quantify this difference, we identified the transition point between the soma and the dendrite in each dataset and quantified the signal intensity in each compartment separately (Fig. 4A). We found that in control fish, the mean signal intensity in somas was 19.1 ± 0.803 [AU], lower than the mean intensity in the dendrites: 19.9 ± 0.89 [AU] (*N* = 8 M cells; Fig. 4B; signed rank = 0, *P* = 0.0078 according to a Wilcoxon signed-rank test). Moreover, in no control M cell was the signal intensity in the dendrite lower than in the soma. We next asked whether the same intensity difference existed in experimental larvae, or, alternatively, whether the increase in the GlyR signal in these larvae was non-uniform between compartments. We found that also in experimental larvae, GlyR signal intensity was slightly higher in dendrites than in somas (somas: 26.15 ± 0.95 [AU], dendrites: 27.45 ± 0.95 [AU]; *N* = 14; Fig. 4C), a significant difference (signed rank = 5, *P* = 0.0012), even though in this population 2 M cells showed an opposite trend. To compare the extent of signal increase in experimental larvae and controls, we computed the dendrite-to-soma GlyR signal intensity ratio for each M cell and fish. Thus, we found the ratio to be above 1 in a statistically significant manner in both controls (1.05 ± 0.01; t = 4.475, df = 5, *P* = 0.0065; two-tailed *t*-test with unequal variances) and experimental larvae (1.06 ± 0.01; t = 4.775, df = 7, *P* = 0.002; Fig. 4D), with no significant difference between the groups (t = 0.4, df = 11.86, *P* = 0.6947; two-tailed *t*-test with unequal variances). These results imply that GlyR density is consistently higher in the lateral M cell dendrite than in the soma, and that post-seizure, GlyR density increases uniformly in these compartments, such that the dendrite-to-soma density ratio remains unchanged.

### Blocking GlyRs with strychnine, which induces synapse disassembly, restores neural responses to startling stimuli

Having identified a decrease in neural responses to startling stimuli and spontaneous neural activity, as well as an increase in GlyR densities at glycinergic synapses following a PTZ-induced seizure, we next asked whether reversing the increase in GlyR densities would also reverse the post-seizure impact on neural activity. Thus, we repeated the seizure induction with 15 mM PTZ application for 1 h, adding a 1 h treatment with 300 μM strychnine, previously demonstrated to cause disassembly of GlyR clusters in glycinergic synapses on the M cells.^67^ Since in these experiments neural activity was examined 75 min post-seizure rather than after 40 min as in experiments described above (Figs 1 and 2; details in the Materials and Methods section), we further added a control group that underwent 15 mM PTZ application and to which E3 was subsequently applied instead of strychnine. Thus, in this group neural activity was examined at the same post-seizure time as in the PTZ + strychnine group.

**Figure 5:**
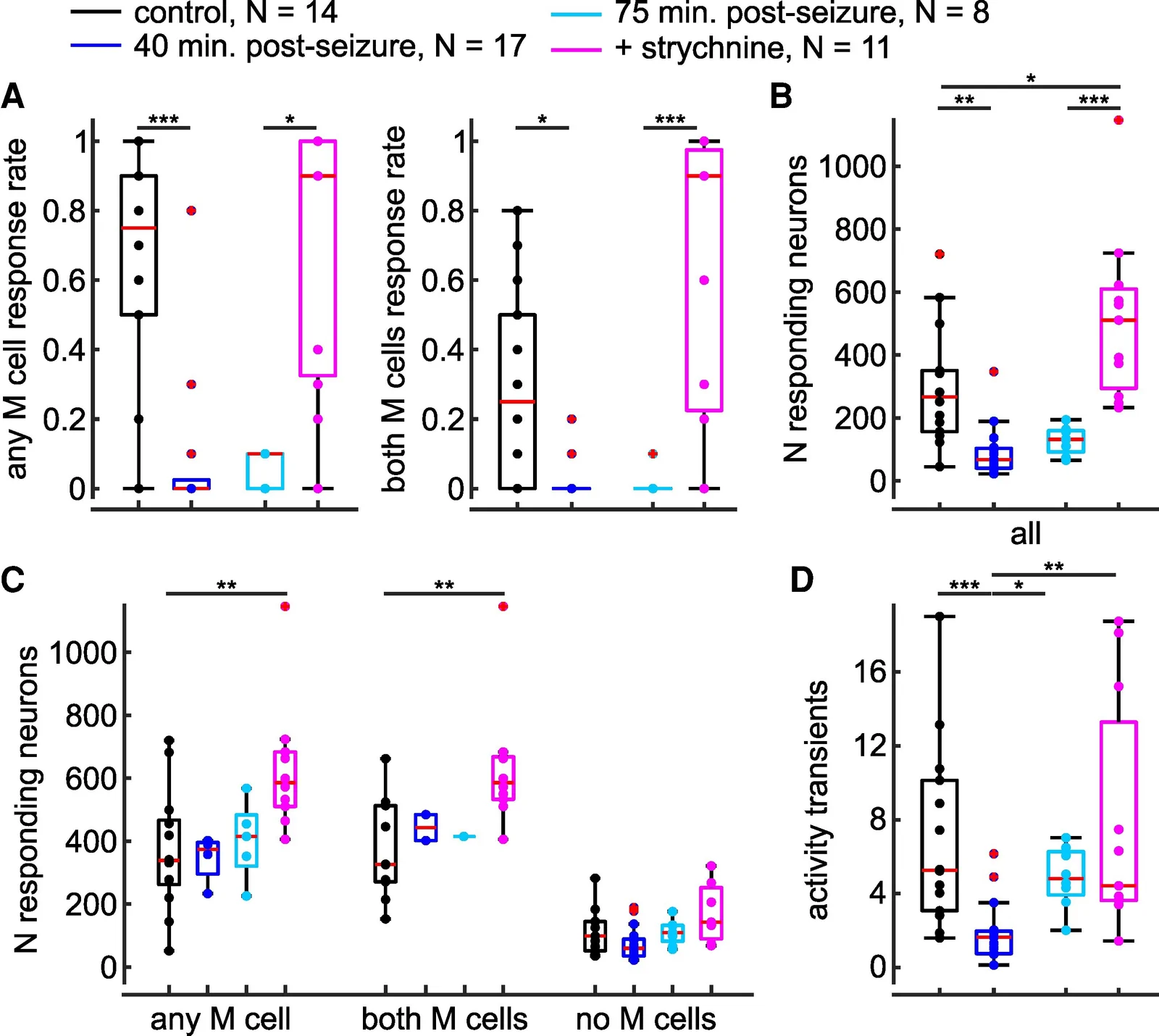
**Post-seizure strychnine application recovers neural responsivity to startling stimuli.** (**A**) Box plots of the fraction of trials in which a response in any of the M cells (left) or both of them (right) was observed, in controls (black boxes, *N* = 14 fish), post-seizure larvae examined 40 min post-seizure (blue boxes, *N* = 17 fish), and 75 min post-seizure (cyan boxes, *N* = 8 fish) or post-seizure larvae to which strychnine was applied for 1 h (magenta boxes, *N* = 11 fish). Colored dots in this and all figure panels represent values from individual fish. *** *P* < 0.001; * *P* < 0.05 according to post-hoc multiple comparisons using the Dunn-Šidák method following a Kruskal-Wallis test which showed a significant effect to the condition (df = 3, χ^2^ = 28.28 and df = 3; χ^2^ = 28.32 for the response rates of any or both M cells, respectively). (**B**) Box plots of the number of hindbrain neurons that responded to the stimulus, in the same experimental groups as in A. *** *P* < 0.001; ** *P* < 0.01; * *P* < 0.05 according to post-hoc multiple comparisons using the Tukey-Kramer procedure following a 1-way ANOVA analysis which showed a significant effect to the condition (df = 3,46, F = 16.4). (**C**) Box plots of the number of hindbrain neurons that responded to the stimulus, in the same experimental groups as in A, divided by whether or not any (left), both (middle) or no (right) M cells were active in the corresponding trials * *P* < 0.05 according to post-hoc multiple comparisons using the Tukey-Kramer procedure following a 2-way ANOVA analysis which showed a significant effect to both the M cell responsivity (df = 3,86; F = 11.04) and the condition (df = 2,86; F = 50.73). (**D**) Box plots of the mean number of activity transients across all neurons in the same experimental groups as in A. *** *P* < 0.001; ** *P* < 0.01; * *P* < 0.05 according to post-hoc multiple comparisons using the Dunn-Šidák method following a Kruskal-Wallis test, which showed a significant effect to the condition (df = 3, χ^2^ = 20.68).

Interestingly, inducing glycinergic synapse disassembly with strychnine application after the PTZ-induced seizure indeed reversed the reduction of neural activity in response to startling stimuli (Fig. 5). In particular, in larvae that underwent strychnine treatment after the PTZ-induced seizure, at least one M cell responded to startling stimuli in 69.1 ± 11.6% of trials (*N* = 11); a higher response rate than in the corresponding 75 min. post-seizure group, 6.3 ± 1.8% (*N* = 8) and similar to the rate in control larvae that did not undergo any treatment, 65 ± 8.6% (*N* = 14; Fig. 5A, **left**). Applying a Kruskal-Wallis test to these response rates, there was a significant effect to the condition (df = 3, χ^2^ = 28.28, *P* = 3.17254 × 10^−6^) and post-hoc tests revealed a significant difference between the PTZ + strychnine group and the corresponding post-seizure group (z = 3.02, *P* = 0.015) but not between the PTZ + strychnine group and the untreated controls (z = 0.36, *P* = 0.9995). In addition, there was no difference in response rates between the 75 min and 40 min post-seizure groups (7.7 ± 4.9%, *N* = 17, z = 0.58, *P* = 0.993). The same trend was evident in the rate at which both M cells responded (Fig. 5A, **right**), where there was also a significant effect of the condition (df = 3; χ^2^ = 28.32; *P* = 3.1067 × 10^−6^). In particular, in the PTZ + strychnine group, both M cells responded in 63.6 ± 11.7% of trials, a much higher rate than in the corresponding post-seizure group, 1.3 ± 1.3% (z = 3.93, *P* = 5.0579 × 10^−4^) but not significantly different than the rate in untreated controls (29.3 ± 7.45%, z = 1.78, *P* = 0.3708). Again, there was no significant difference between the post-seizure groups examined at distinct post-seizure times (1.8 ± 1.3%, z = 0.02, *P* = 1). Since M cell activity rates did not differ in the two post-seizure groups, we repeated the analysis while merging these groups into one group (Supplementary Fig. 3A). This did not affect the above described results demonstrating a significant effect to the condition (any M cell response rates: df = 2, χ^2^ = 27.95, *P* = 8.547 × 10^−7^; both M cells response rates: df = 2, χ^2^ = 28.32, *P* = 7.076 × 10^−7^) as well as significant differences between the PTZ + strychnine and the pooled post-seizure group (any M cell response rates: z = 4.35, *P* = 8.298 × 10^−5^; both M cells response rates: z = 5.03, *P* = 2.9069 × 10^−6^) and not the control groups (any M cell response rates: z = 0.36, *P* = 0.9995; both M cells response rates: z = 1.78, *P* = 0.3708).

Having observed the recovery of post-sseizure M cell responses following strychnine application, we next examined the responsivity of hindbrain neurons to the stimulus more broadly. Examining the total number of neurons that responded to the stimulus in PTZ + strychnine larvae compared to PTZ-treated larvae and untreated controls (Fig. 5B), we again found, using a 1-way ANOVA, a significant effect of the condition (df = 3,46, F = 16.4, *P* = 2.6262 × 10^−7^). Moreover, post-hoc tests showed that the number of responding neurons in the PTZ + strychnine group (513 ± 80) was significantly higher than both the number of responding neurons in the corresponding post-seizure group (128 ± 16, q = 7, *P* = 5.966 × 10^−5^) as well as the number of responding neurons in untreated controls (302 ± 51, q = 4.42, *P* = 0.0157). Again, there was no significant difference between the post-seizure groups examined at distinct post-seizure times (40 min. post-seizure: 89 ± 19, q = 0.7593, *P* = 0.9496). Repeating the analysis while merging the non-different post-seizure groups (Supplementary Fig. 3B) did not change the results, the difference between the overall number of responding neurons in the PTZ + strychnine group was again significantly higher than in the post-seizure group (q = 9.68, *P* = 4.2997 × 10^−8^) as well as the number in the control group (q = 4.54, *P* = 0.0079) according to post-hoc multiple comparisons following a 1-way ANOVA which showed a significant effect to the condition (df = 2,49, F = 24.44, *P* = 5.2874 × 10^−8^).

Next, we examined the dependence of the number of responding neurons on M cell activity, in the presence of strychnine (Fig. 5C). As before including the post-seizure strychnine application, conducting a 2-way ANOVA analysis to identify whether the number of responding cells depended on whether or not the M cells responded, or on the condition, or both, showed a significant effect to M cell responsivity (df = 3,86; F = 11.04; *P* = 3.3448 × 10^−6^). However, in contrast to the result without the strychnine treatment, this analysis now also showed an additional effect of the condition (df = 2,86; F = 50.73; *P* = 2.8073 × 10^−15^). Using post-hoc multiple comparisons to identify the groups that significantly differed, we found that the number of responding neurons after a PTZ-induced seizure followed by strychnine treatment was significantly higher than the number in untreated controls both when at least one of the M cells was active (PTZ + strychnine: 630 ± 65; untreated controls: 366 ± 53; q = 6.4, *P* = 0.0011) as well as when both were active (PTZ + strychnine: 633 ± 63; untreated controls: 371 ± 50, q = 5.96, *P* = 0.0034); but not when none of the M cells were active (PTZ + strychnine: 173 ± 36; untreated controls: 114 ± 22; q = 1.25, *P* = 0.9991). However, there was no significant difference between the number of responding neurons in the strychnine treatment group and the corresponding post-seizure group, independent of M cell activity, likely due to the small number of trials in which M cells responded in the post-seizure group, giving rise to a high uncertainty in these results (for the post-seizure group, the number of responding neurons for at least one active M cell: 403 ± 57, q = 4.22, *P* = 0.1313 compared to the strychnine treatment group; when both M cells were active: 415 ± 0; q = 2.12, *P* = 0.9371; when no M cells were active: 110 ± 14; q = 1.25, *P* = 0.9991). When merging the post-seizure groups together (Supplementary Fig. 3C), as they showed no significant differences (any M cells active—q = 0.87, *P* = 1; both M cells active—q = 0.23, *P* = 1; no M cells active—q = 0.87, *P* = 1) the difference between the strychnine group and the merged PTZ group when at least one M cell was active was statistically significant according to a post-hoc test following a 2-way ANOVA analysis (q = 5.67, *P* = 0.0039), but the difference when both M cells were active remained insignificant (q = 3.12, *P* = 0.4093). Put together, this analysis shows that strychnine treatment after a PTZ-induced seizure elevates neural responsivity to the stimulus not only by increasing the fraction of trials in which M cells respond to the stimulus, but also independently of this effect, and in particular in trials in which the fish decided to escape.

Finally, we also compared the number of spontaneous activity transients between the post-seizure strychnine-treated group, the two post-seizure groups and untreated controls (Fig. 5D). Since the number of peaks in both the 40 min. post-seizure as well as in the PTZ + strychnine group across larvae were not normally distributed, we used a non-parametric approach in the analysis of this data. Using a Kruskal-Wallis test to examine the number of spontaneous activity transients we found it to be significantly affected by the condition (df = 3, χ^2^ = 20.68, *P* = 1.2278 × 10^−4^). Using post-hoc tests to examine which groups significantly differed, we found, interestingly, that the number of spontaneous activity transients in the 75 min. post-seizure group (4.88 ± 0.58), without the added strychnine treatment, already indicated recovery, and was not significantly different than the number in untreated controls (6.98 ± 1.32, z = 0.28, *P* = 0.999). In addition, strychnine treatment did not significantly increase the number of spontaneous activity transients beyond its level in this corresponding post-seizure group (7.84 ± 1.92, z = 0.25, *P* = 0.9999) or in untreated controls (z = 0.03, *P* = 1). The number of spontaneous activity transients in the earlier, 40 min. post-seizure group (1.85 ± 0.39), was thus significantly reduced compared to the number of transients in all other groups (untreated controls: z = 3.85, *P* = 7.1179 × 10^−4^, 75 min. post-seizure: z = 2.95, *P* = 0.0192, PTZ + strychnine: z = 3.56, *P* = 0.0022). We note that since in this case there was a significant difference between the two post seizure groups, we did not re-analyse the data while pooling these groups together in this case. Put together, these data show that the post-seizure decrease in spontaneous neural activity is transient, and recovers by 75 min post-seizure, in contrast to the decrease in stimulus responsivity, which lasts longer. Together with the absence of an effect of strychnine treatment on the number of spontaneous activity transients, this suggests that the post-seizure effects on stimulus-induced and spontaneous activity may be mediated by distinct mechanisms.

## Discussion

Due to their impact on brain structure and function, seizures are thought to have a causal role in giving rise to cognitive impairment and neuronal deficits in epilepsy, also caused by genetic mutations underlying the disease.^11-15^ Moreover, they may be associated with an increase in seizure susceptibility. Thus, understanding how seizures impact the brain is key for explaining the diversity of cognitive deficits in epilepsy and potentially mitigating them. We used the amenability of the larval zebrafish epilepsy model for non-invasive *in vivo* neural activity imaging, combined with the advent of ExM for high-resolution synaptic imaging, to reveal brain functional and structural alterations caused by acutely induced seizures in wild-type animals, in the absence of confounding factors. Thus, we found that M cell responses to startling stimuli were markedly reduced after seizures—pointing to a reduced rate in which larvae decided to escape.^60^ Moreover, both neural activity in response to startling stimuli, as well as spontaneous activity, were reduced. However, the reduction in spontaneous activity was transient and presented recovery by 75 min post-seizure. In addition, we revealed a post-seizure synaptic modulation and specifically, a compensatory enhancement, in the form of increased receptor densities, as well as an increase in the pre-synaptic signal from the synaptic vesicle protein 2, in glycinergic inhibitory synapses that are not impaired during the seizure. Glycinergic synapses have an established role in setting the threshold of M cell activation.^45,64,68^ Thus, this enhancement likely contributes to the reduction in the responses of M cells and other neurons to the stimuli. Interestingly, following a 1 h-long application of strychnine, which blocks glycinergic synapses and was previously shown to cause their disassembly,^67^ the post-seizure effect on M cell responses specifically, and neural responsivity to the stimulus more broadly, was reversed. This result suggests that the increase in glycine receptor densities, strengthening glycinergic inhibitory synapses, plays a causal role in mediating the observed post-seizure phenotypes. Finally, since the general decrease in neural activity we observed was more transient than the reduced neural responsivity to startling stimuli, and as it was not affected by strychnine application, we conclude that the decrease in spontaneous activity was likely mediated by a distinct mechanism.

A decrease in sensory sensitivity and neural activity, as shown here, within an hour of an induced seizure, is consistent with the definition of the post-ictal state in humans.^8,9^ Post-ictal behavioural deficits were also observed in rodent models,^69-71^ and studies in both humans and rodents suggest that post-ictal phenotypes arise from altered neural processing in seizure affected areas.^72,73^ We recently showed that startle behaviours of 6 dpf larval zebrafish are transiently suppressed, for up to 6 h, after a 1 h-long seizure in 15 mM PTZ,^74^ consistent with the reduction in post-seizure neural responsivity to these stimuli presented in this work. Moreover, this suggests that the effect observed here, of reduced neural responsivity to startling stimuli, is also transient at this time-scale, which is longer than the time-scale of 75 min post-seizure in which we observed recovery of spontaneous neural activity. In addition, our current results suggest that the post-ictal decrease in neural and behavioural sensory responsivity is mediated by enhancement of inhibitory glycinergic synapses for a seizure induced by blocking other inhibitory, in particular GABAergic, synapses. A similar enhancement of glycinergic synapses via increased receptor densities, observable as higher fluorescent signal in the synapses, was previously reported as a mechanism of non-associative learning, specifically desensitization leading to reduced startle behaviour and M cell responses.^64,75^

Both the enhancement driven by non-associative learning and the enhancement we observe here could be mediated by rapid glycinergic synapse modulation via the net movement of receptors from extrasynaptic spaces into synapses, due to an activity-dependent increase in glycine receptor affinity to the scaffold protein gephyrin.^76^ This mechanism allows changes in glycinergic synapse strength to occur at short time scales.^77^ Nevertheless, the enhancement we observed also involved the presynaptic compartments, which were not analysed in the desensitization study. Moreover, we found no change in the fraction of the M cell membrane covered by receptors—indicating that synaptic sizes or numbers did not change, but rather the synapses are specifically strengthened by increased receptor densities. Whether non-associative learning-induced changes in glycinergic synapses involve the same increase in density or increased sizes or numbers of synapses is unknown. Post-ictal enhancement of inhibitory glycinergic synapses is also consistent with several studies that examined seizure- or epilepsy-related changes in inhibitory synapses. E.g., one study in rodents showed that inhibition through GABA_B_ receptors, but not GABA_A_ receptors, mediates post-ictal refractoriness minutes after seizure termination.^78^ Moreover, an increase in expression of high-affinity GlyRs was identified in the hippocampi of severe temporal lobe epilepsy patients.^79^ In addition, PTZ-induced seizures were also previously associated with reduced excitatory synaptic transmission 1 day after the seizure,^80^ and while we did not find changes in the pre-synaptic SV2 signal outside of the vicinity of glycinergic synapses, we cannot rule out that reduced excitatory transmission also contributes to the reduced neural activity and responsivity we observed.

Similar to our finding of post-seizure neural activity modulation, studies in rodent models reveal diverse post-seizure neural activity modulations across a wide range of post-seizure timelines, explaining both immediate and long-term cognitive deficits caused by seizures. These included altered functional connectivity, decreased power of theta oscillations, decreased synchronization of specific neural populations, diminished coherence of activity in different brain regions, as well as reduced precision and stability of hippocampal spatial fields, associated with reduced spatial memory.^11,81-84^ The modulation we observed is also consistent with previously reported reduced inter-ictal neural activity and behaviour in a genetic larval zebrafish epilepsy model.^85^ Furthermore, similarly to our observation of reduced activity and sensory responsivity following seizure-induced hyperactivity, it was found that both during PTZ application, as well as in genetic larval zebrafish models of epilepsy, elevated activity induced by a photic stimulation is followed by a depressed state.^86^

Post-seizure PTZ effects were also previously studied in adult zebrafish, revealing modified behaviours: impaired learning, memory and social shoaling, as well as increased anxiety and aggression.^87-90^ Some of these modulations occurred within 1 h post-seizure, while others occurred 24–48 h later and recovered by 72 h post-seizure. The structural and functional modulations we observe, at a short time scale, may underlie, or initiate functional changes leading to, such behavioural modulations. In addition to these behavioural modulations, neurotransmitter levels were found to be altered immediately after PTZ-induced seizures, at the same time in which we observe structural and functional modulations. Specifically, post-seizure dopamine and serotonin (5-HT) levels were higher in experimental larvae compared to controls, while GABA, glutamate and acetylcholine levels were lower.^91,92^ One study in larval zebrafish found that global increases in neural activity, including those induced by PTZ but not limited to seizure activity, give rise to a rebound sleep state mediated by activity in neurons releasing the neuropeptide galanin.^93^ This is consistent with our finding of reduced post-seizure neural activity, suggesting the involvement of the same mechanism in our observations as well. However, responses to strong stimuli such as the escape-inducing taps we applied are expected to be intact during a period of rebound sleep due to the reversibility of the sleep state, in support of an additional synaptic mechanism, as we found, mediating this additional functional effect. Finally, a study in larval zebrafish showed a structural effect, a reduction in cell proliferation, 24 h after seizure induction by PTZ or other convulsants.^94^ Thus, our studies extend existing evidence for structural and functional modulations following PTZ-induced seizures in zebrafish, demonstrating that such changes also occur in larvae, involve synaptic modulation, and are already present within an hour of the seizure termination.

Comparing these results to studies in rodents, a single PTZ-induced seizure was shown to cause cognitive, and specifically, learning and memory deficits. Some of these deficits were present 1 h after seizure,^95^ others one or a few days later,^81,96^ and some only after multiple weeks.^97,98^ In contrast, structural modulations such as enhanced neurogenesis, increased proliferation and intracellular organelle degeneration were observed as early as 1 day post-seizure, and neuronal damage and death were observed immediately and up to one month after seizures.^97,99-103^ Synaptic modulations were also observed, including a decrease in the probability of glutamate release as well as modulation of long-term potentiation, within minutes or hours after a PTZ-induced seizure.^12,104-106^ Similarly, status epilepticus induced by other convulsants such as pilocarpine or kainic acid, or by electrical excitation, gave rise to learning and memory impairments^81,107,108^ and to structural modulations including cell loss and damage,^109-112^ synaptic modulations,^12,113-115^ and aberrant neurogenesis or circuit rewiring.^109,116-118^ Some of the results associated with application of kainic acid and pilocarpine were also replicated in adult zebrafish.^119-121^

All of the findings described above are broadly consistent with our observations of altered neural function and synapse structure after an induced seizure. However, interestingly, while status epilepticus induced by either pilocarpine or kainic acid was found to lead to epileptogenesis—the delayed emergence of spontaneous, recurrent seizures,^16,122,123^ this is not the case for PTZ-induced status epilepticus.^12^ This difference in the effects of status epilepticus induced by distinct mechanisms is consistent with reports of reduced glutamate release probability after PTZ-induced seizures in rodents, and with our observation of reduced neural activity and strengthened inhibitory glycinergic synapses following a PTZ-induced seizure in larval zebrafish. Moreover, modulations of inhibitory circuit motifs in epileptogenesis models, even when involving strengthening of some inhibitory modules, also included loss of inhibitory neurons and synapses as well as rewiring that reduced inhibition, such that overall there was a net increase in excitation that subsequently mediated spontaneous seizures.^114,124,125^ Put together, this suggests that the enhanced synaptic inhibition and reduced neural activity and sensory responsivity we observed may represent an anti-epileptogenic mechanism and its effect in the larval zebrafish PTZ-induced seizure model. Future comparisons of these effects to deficits in other larval zebrafish seizure models, including genetic epilepsy models, particularly ones in which seizures can be triggered by sensory stimulation, making the post-seizure period easy to define,^85,126^ could further shed light on mechanisms contributing to protection from epileptogenesis.

## Supplementary Material

fcag323_Supplementary_Data

## Data Availability

The data that support the findings of this study are available from the corresponding author upon request. The code that was used for neural activity imaging and ExM data analysis can be freely downloaded from the Open Science Framework using this link: https://osf.io/sd5jy/overview?view_only=996dc66dbec64cabaf5db26496197fca.
