# Supplementary material for: Induced epileptic seizures in larval zebrafish reveal a synaptic modulation associated with the post-ictal state
Source: Brain Commun. 2026 Sep 8;8(5):fcag323. doi: 10.1093/braincomms/fcag323 (PMC13550772; doi:10.1093/braincomms/fcag323)
Supplement: Supplementary file 1 [file fcag323_supplementary_data.pdf]

Supplementary Figures

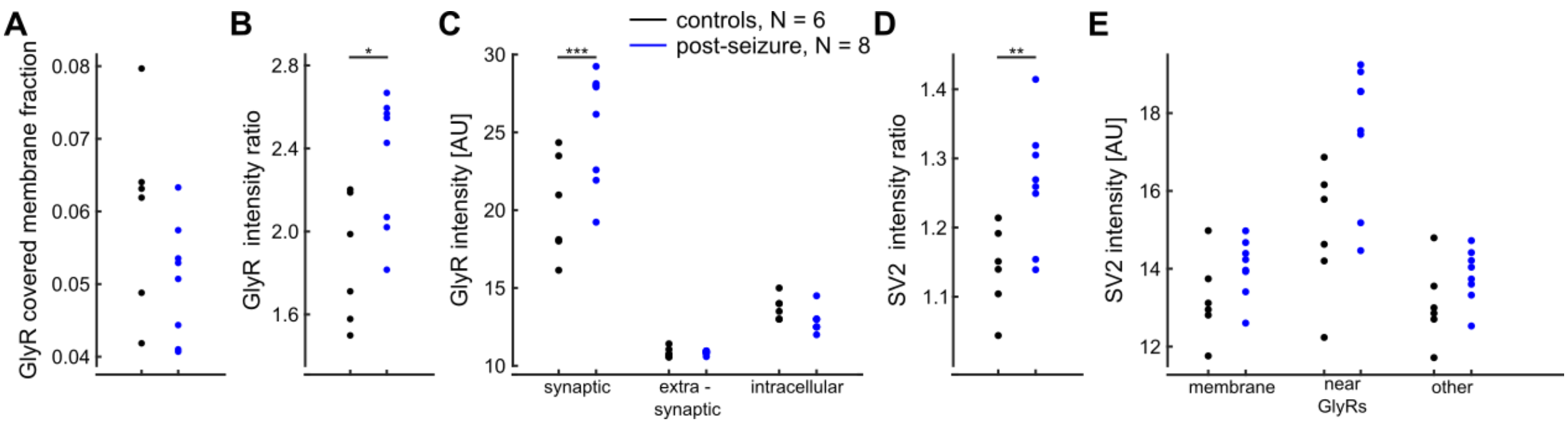

Supplementary Figure 1. Enhancement of glycine receptors (GlyR) and Synaptic-vesicle protein 2 (SV2) signals at glycinergic synapses. A-E. Same as in Fig. 3C-G, showing individual sample points rather than box plots.

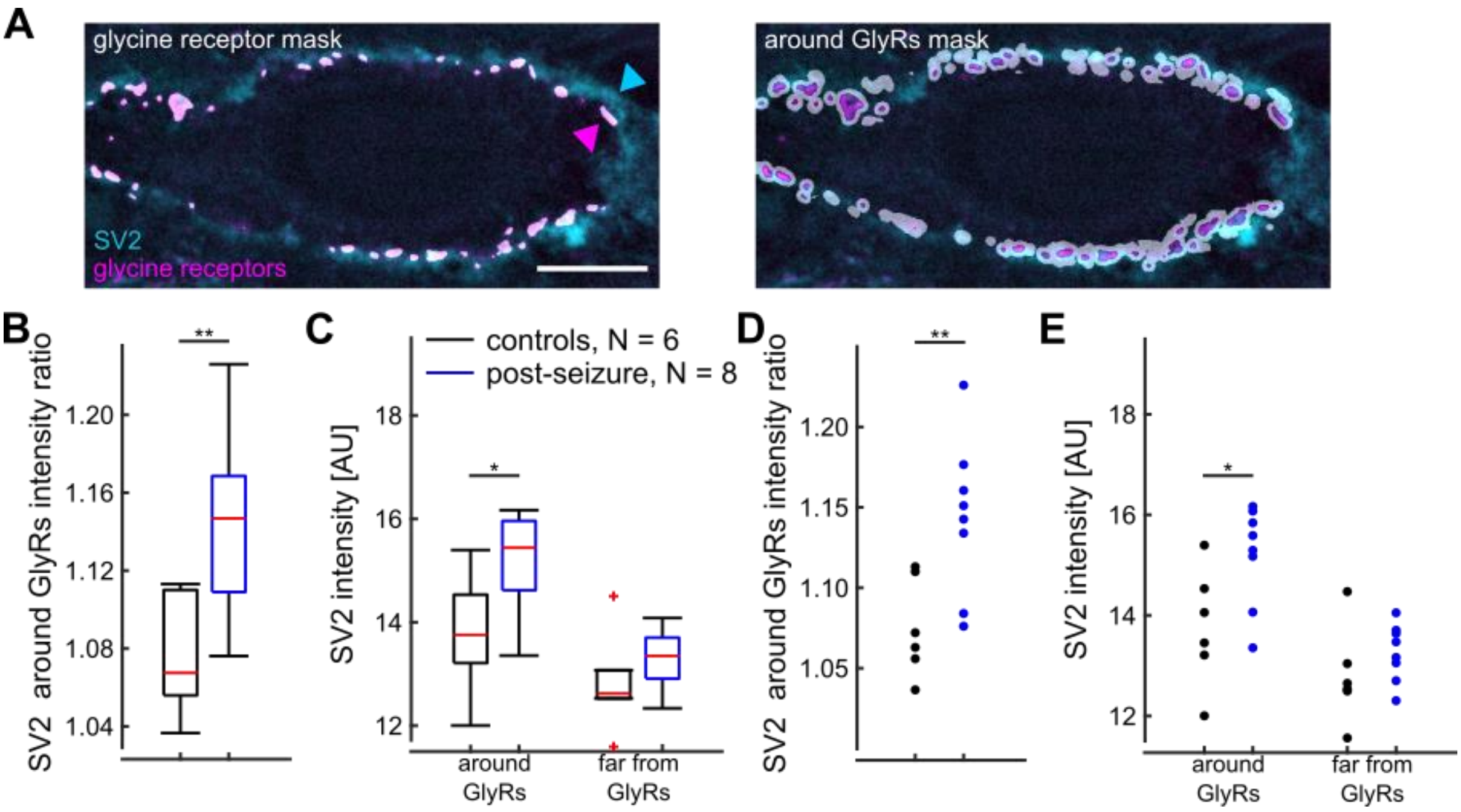

Supplementary Figure 2. Pre-synaptic enhancement apparent as SV2 signal increase around GlyRs. **A.** Zoom-in on a part of the plane shown in Fig. 3B, showing the pre-synaptic SV2 signal (cyan and cyan triangle) slightly shifted away from the center of the M cell with respect to the GlyR signal (magenta and magenta triangle). The image is overlaid with a segmentation mask of the GlyRs within the membrane (top), or their surroundings (bottom, “around GlyR mask”). **B.** Box plots describing the ratio of SV2 intensity in around GlyRs (segmented as shown in A, top) and the SV2 intensity far from GlyRs (not included in either the GlyR mask or the “around” GlyR mask shown in A) in controls (black box) and experimental fish (blue box). ** $p < 0.01$ according to a two-tailed t-test with unequal variances (t(degrees of freedom – df = 11.77) = 3.25, SE = 0.0212). **C.** Box plots describing the SV2 intensity around segmented synapses (left) and in the rest of the M cell membrane –excluding both pixels within the synapses as well as ones around the synapses (right), for controls (black boxes) and experimental (blue boxes) fish. * $p < 0.05$ according to post-hoc multiple comparisons using the Tukey-Kramer’s procedure following a two-way ANOVA analysis which identified a significant effect to the position (around or far from GlyR, F = 16.62; df = 1,24) as well as to the condition (experiment vs control, F = 7.1; df = 1,24). **D-E.** Same as B-C, respectively, showing individual samples (means per fish) rather than box plots.

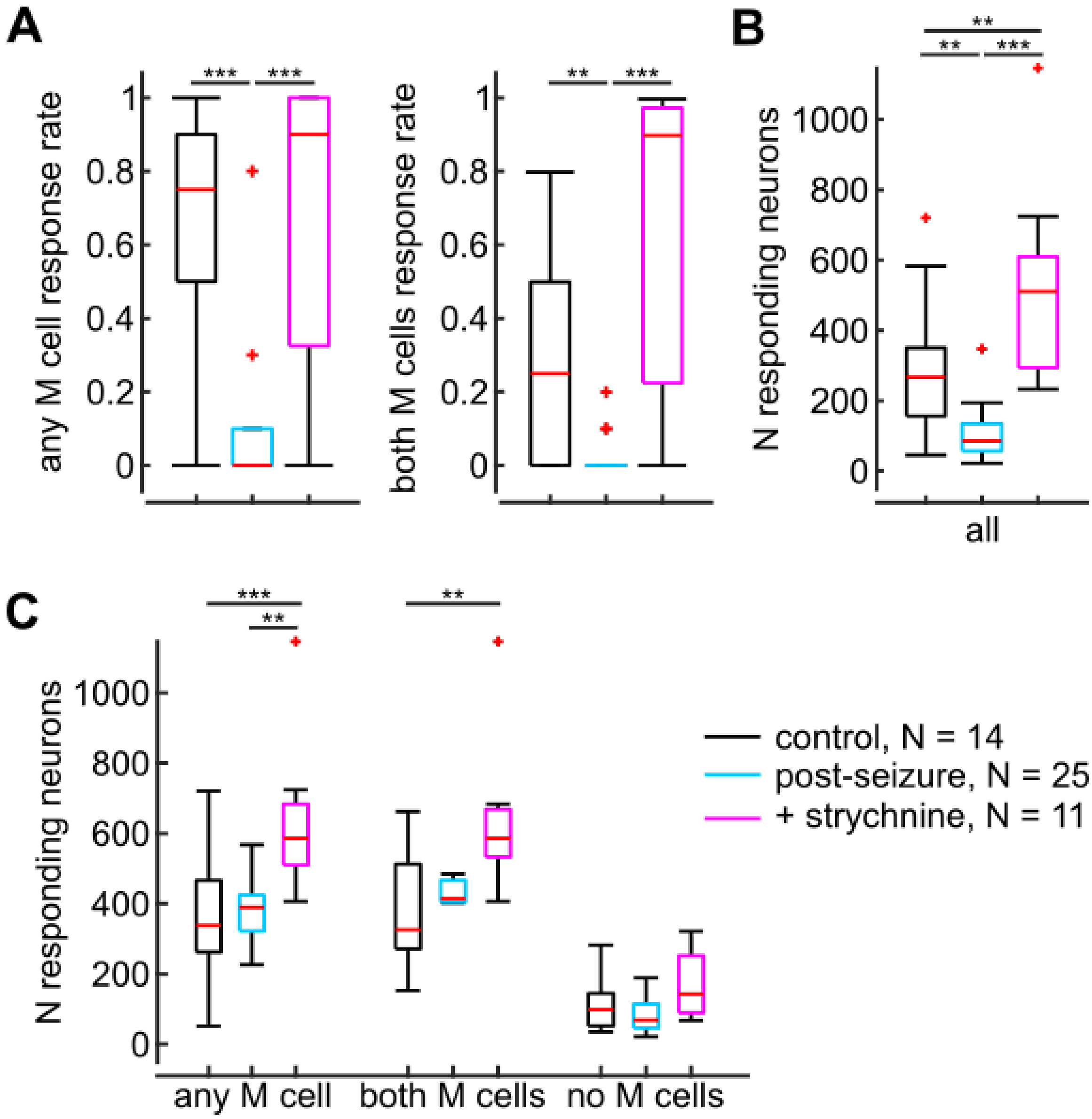

Supplementary Figure 3. Post-seizure Strychnine application increases neural responsivity to startling stimuli. A. Box plots of the fraction of trials in which a response in any of the M cells (left) or both of them (right) was observed, in controls (black boxes, N = 14 fish), post-seizure larvae examined 40 or 75 minutes post-seizure (cyan boxes, N = 25 fish) or post-seizure larvae to which strychnine was applied for 1 hour (magenta boxes, N = 11 fish) *** $p < 0.001$ according to post-hoc multiple comparisons using the Dunn-Šidák method following a Kruskal-Wallis test which showed a significant effect to the condition both on the response rate of any of the M cells (df = 2, $\chi^2 = 27.95$) as well as on the response rate of both M cells (df = 2, $\chi^2 = 28.32$). **B.** Box plots of the number of hindbrain neurons that responded to the stimulus in the same groups as in A. *** $p < 0.001$; ** $p < 0.01$ according to post-hoc multiple comparisons using the Tukey-Kramer procedure following a 1-way ANOVA analysis which showed a significant effect to the condition (df = 2,49, F = 24.44). **C.** Box plots of the number of hindbrain neurons that responded to the stimulus, in the same experimental groups as in A, divided by whether or not any (left), both (middle) or no (right) M cells were active in the corresponding trials *** $p < 0.001$; ** $p < 0.01$ according to post-hoc multiple comparisons using the Tukey-Kramer procedure following a 2-way ANOVA analysis which showed a significant effect to both the condition (df = 2,89, F = 16.81) and to the M cell responsivity (df = 2,89, F = 60.7).
